# Effects of Intrathecal Opioids Use in Cesarean Section on Breastfeeding and Newborns’ Weight Gaining 

**Published:** 2016-12

**Authors:** Fardin Yousefshahi, Fatemeh Davari-Tanha, Atabak Najafi, Mahbod Kaveh, Mohsen Rezaei Hemami, Patricia Khashayar, Mohammad Anbarafshan

**Affiliations:** 1 Department of Anesthesiology, Imam Khomeini Hospital Complex, Tehran University of Medical Sciences, Tehran, Iran; 2 Brain and Spinal Cord Injury Research Center, Neuroscience Institute, Tehran University of Medical Sciences, Tehran, Iran; 3 Department of Obstetrics and Gynecology, Women’s Hospital, Tehran University of Medical Sciences, Tehran, Iran; 4 Department of Anesthesiology, Anesthesia and Critical Care Department, Sina Hospital, Tehran University of Medical Sciences, Tehran, Iran; 5 Department of Pediatrics, NICU Department, Women’s Hospital, Tehran University of Medical Sciences, Tehran, Iran; 6 Department of Epidemiology, Rajaie Cardiovascular, Medical and Research Center, Iran University of Medical Sciences, Tehran, Iran; 7 Endocrinology and Metabolism Research Center, Tehran University of Medical Sciences, Tehran, Iran

**Keywords:** Breastfeeding, Cesarean Section, Intrathecal Opioid, Spinal

## Abstract

**Objective:** To assess the association between intrapartum intrathecal opioid use and breastfeeding and weight gain following cesarean section.

**Materials and methods:** The prospective double-blinded study was conducted on term pregnant women, undergoing elective cesarean section under spinal anesthesia. They divided into two groups. In the first group, intrathecal Morphine was used to achieve analgesia during or after the operation. The remainder divided into two subgroups, those who did not receive any opioid or those received systemic opioids. Following labor breastfeeding accessed in a follow-up, two month latter.

**Results:** There was no difference between the demographic variables of the mothers and newborns APGAR score and weight at the time of birth. Breastfeeding rate was similar in intrathecal group in compare with other patents (P value = 0.518). While, the infants’ weight at the end of second month was lower in spinal opioid group (P value = 0.036).

**Conclusion:** The present study was the first to suggest that spinal (intrathecal) opioids do not have any impact on breastfeeding. However the relationship between spinal anesthesia on weight gaining needs more investigation.

## Introduction

Breastfeeding difficulties in the early postpartum period, especially if combined with inadequate support and advice, may cause new mothers give up nursing their babies.

Many factors, including demographic, biological, social, and psychological variables are influencing breastfeeding. Race, age, marital status, education, socioeconomics, and supplemental nutrition programs, maternal obesity, physical challenges of breastfeeding like sore nipples, engorgement, milk insufficiency, maternal smoking, parity, delivery method, occupation, family and professional supports are all known to affect breastfeeding process and pattern. Several other factors like; maternal intention, interest, and confidence to breastfeeding, parity, infant status, poor newborn weight gain, difficulty of the newborn in latching onto the breast or sucking, infant being demand fed in hospital, discontented baby as well as the type of labor and birth, and even other family member preference for breastfeeding may influence the establishment of breastfeeding (1-6).

In addition, the literature has identified the use of pain medication by the mother during labor to influence breastfeeding success (7, 8). There are controversial reports regarding the effects of pain relief medication on neonatal breastfeeding duration (7-12).

Initial studies in this regard, however, mainly focus on the impact of epidural opioid analgesics on the newborn’s growth rate and neurobehavioral; which are considered to have a significant influence in reducing the breastfeeding rate (13-16). However more recent studies are discussing safety of regional anesthesia and intrathecal opioids on breastfeeding (17-19). 

Considering the fact that the majority of the available studies have focused on women receiving epidural analgesics, and regarding the popularity of spinal anesthesia to perform cesarean section; this study aimed to determine whether there is any association between intrathecal opioid use on breastfeeding following cesarean section under spinal anesthesia.

## Materials and methods

Approved by the Ethical Board Committee of Tehran University of Medical Sciences, ethical number 7714-300188, this prospective double-blinded study was conducted in Women Hospital (certified as Baby Friendly Hospital Initiative (BFHI) -- a worldwide program of the World Health Organization and UNICEF --). All term (gestational age ≥ 37 week) pregnant women undergoing elective or emergency Cesarean Section under spinal anesthesia who had consent to be included in the study, and hadn’t received opioids within a month before the procedure were included.

Patients with previous history of head trauma in need of hospital administration or neurologic disorders along with those experiencing severe pre-eclampsia, and other medical conditions leading to Intrauterine Growth Retard (IUGR) as well as those who had been treated for infertility were excluded from the study. Mothers who had a history (even a suspicious history) of addiction, alcohol or stimulant drug consumption, smoking history, along with those taking anti-convulsive medication, antidepressants and tranquillizers were also excluded from the study. Women with endocrinologic disorders, history of breast anomalies and/or surgery as well as other underlying causes such as a history of postpartum depression interfering with breastfeeding were also excluded. Women with a previous history of breast operation or suffering from any surgical or anesthesiologic complication and those whose infants were Low Breath Wight (LBW), had a 5’th minute Apgar lower than 8 or admitted to Neonatal Intensive Care Unit (NICU) were excluded from the study. Other probable confounders including; mother’s age, weight, Body Mass Index, gestational diabetes, smoking, occupation, education, Obstetric and pregnancy history, breastfeeding history, number of children, and also infants 1’th and 5’th minutes Apgar, weight, and sex, were recorded.

If the woman received any systemic (intravenous, intramuscular, transcutaneous, or inhalational) anesthetic medications during labor, was no more eligible to participate in the study.

All the patients signed an informed consent before entering the study. The patients were primarily divided into two groups using computerized random software. In the first group, intrathecal Morphine (Morphine Sulfate, Darou Pakhsh Co.) (5µg/kg intrathecaly [minimum 300μg and maximum 500μg]) was used to achieve analgesia during or after the operation. After the operation, however, no systemic, neuraxial or local opioids or even other analgesic non-opioids were administered. Patients who need other analgesic medication considered to be excluded latter.

The control group received no neuraxial or local opioids during or after the operation. In this group, non-opioids including Non- Steroidal Anti-Inflammatory Drugs (NSAID's) or acetaminophen was used as first choice of analgesia. A subgroup of control group who had experienced a resistant pain, had received doses of pethidine (20-50 mg) (Pethidine, Caspian Tamin) or fentanyl (50-100 μg) (Fentanyl Citrate, Caspian Tamin) -only in the operating or recovery room just after operation-, to relive pain. The patients who needed more than 100 mg pethidine because of their excessive pain considered to exclude from the study.

Spinal anesthesia was performed in sitting position using a 27 Gauge Quinke spinal needle (Dr. Japan Co. Ltd) through L4-L5 or L5-S1 levels. In these cases, 12-15 mg hyperbaric Bupivacaine Hydrochloride 0.5% (Marcaine Spinal 0.5%, Heavy, AstraZeneca) was used as anesthetic during the operation.

All the patients received 2 mg midazolam following the clamping of the umbilical cord. No other sedative prescribed in any of the patients. Oxytocin (Oxytip^®^, Caspian Tamin) 30 units in 2 hours was administered in all patients. All other medications (antibiotic prophylaxis) and treatments (out of bed, Foley catheter) were performed according to hospital protocols. The demographic and gynecologic characteristics of the subjects recorded and compared between groups. The newborn was visited by a pediatrician and his/her general condition as well as the 1 and 5 minute APGAR score were assessed. The fact whether he or she was transferred to NICU or neonatal high-risk wards was also recorded.

The conditions which may interfere with the nursing process and the medication taken by the mother were recorded. 

All mothers were unaware of the group to which they were allocated. The mothers were encouraged and taught how to breastfeed their newborns according to BFHI points. Mother and child contact started in operation theater for all mothers. As a routine program, an experienced, educated breastfeeding nursing group, unaware of the objectives of the study, was responsible for teaching the technique through a face-to-face education and follow-up the successful in-hospital breastfeeding. The mothers were then asked at the time of discharge to see whether their baby had any problems with breastfeeding or not. There was an additional assessment of breast-feeding by a member of the team, blinded to group assignment, at 8 weeks postpartum.

The team member called all the mothers at 65-75 days postpartum to determine whether they were still breast-feeding. If the mother was not breast-feeding, she was queried as to whether this was related to difficulty the infant was experiencing with breast-feeding or not. Baby weight, the neonate and mother health condition, used medications, any hospital administration, and the mother’s tendency toward breastfeeding were asked during this call. Individuals experiencing conditions which interfere with breastfeeding, including baby or mother disease or hospital administrations, mastitis and postpartum depression, or any condition that interfere with their normal contact, and mothers who did not like to breastfed their babies considered to be excluded from the study. The consumption of any herbal or home ordered food additives or traditional medication used as breastfeeding supporters was recorded. Those who did not answer to call for 4 repeated calls in next days also were excluded.

***Sample Size:*** Considering the study conducted by Jordan et al, the prevalence of breastfeeding in the control group along with women taking neuraxial and systemic opioids was 32%, 52% and 41% (20). By considering the values for neuraxial opioids in compare with mean values of other two groups, the power analysis demonstrated that a sample size of 128 in each group, which would be sufficient to provide 80% power to detect less than 15% difference between the groups.

***Statistical Analysis:*** The gathered data were analyzed using SPSS software version 17. Data were explored with t tests and crosstabs as appropriate. P-values lower than 0.05 were considered as significant. In a logistic regression model for comparing two groups adjusted on possible confounders, the validity of finding accessed.

## Results

**Figure 1:**
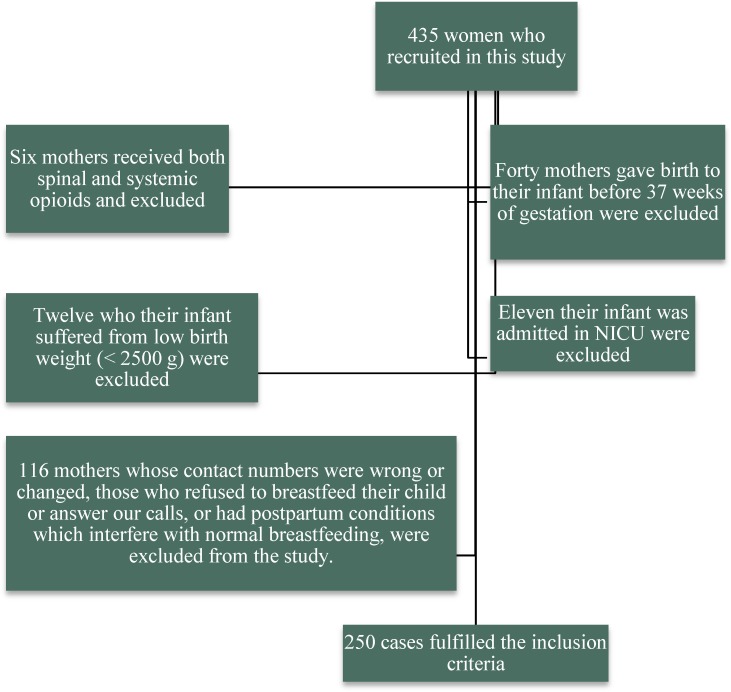
CONSORT chart of the study

From among 435 women who recruited in this study, only 250 fulfilled the inclusion criteria. Mothers who received both spinal and systemic opioids (6), those who gave birth to their infant before 37-40 weeks of gestation , their infant suffered from low birth weight (< 2500 g) (12), or their infant was admitted in NICU (11), and mothers whose contact numbers were wrong or changed, those who refused to breastfeed their child or answer our calls, or had postpartum conditions which interfere with normal breastfeeding, were excluded from the study (n=116) (Figure 1).

We enrolled 250 women with the mean age of 28.07 years, ranging from 17 to 46 years. Table 1 outlines the demographic data of the studied subjects. There was no significant difference between the ages of the studied mothers in the two groups.

About 90.4% of the studied mothers were reported to be housewives. Only 11.3% of the studied mothers had attended college. 29.3% of the subjects were primary gravid, whereas others were experiencing their second to sixth pregnancy. They had between 1 (35.1%) and 4 (0.4%) children. About 47.6% of them had breastfed their older children.

**Table 1:** comparing quantitative variables between spinal and non-spinal groups

**Variables**	**Group**		**P-value**
	**Spinal**	**Nonspinal**	
	**Mean ± (Standard Deviation)**	**Mean ± (Standard Deviation)**	
Mother Age (year)	28.56 ± (5.58)	27.83 ± (5.05)	0.305
Mother Weight (kg)	77.87 ± (13.70)	76.43 ± (12.74)	0.421
Mother Height (cm)	161.51 ± (5.52)	160.24 ± (6.15)	0.122
Mother BMI* (kg/m^2)^	29.80 ± (4.70)	29.83 ± (4.77)	0.960
Gravid	2.10 ± (0.94)	2.22 ± (1.08)	0.401
Para	1.94 ± (0.85)	2.08 ± (0.93)	0.230
Number of Children	0.96 ± (0.88)	1.10 ± (0.94)	0.288
Pregnancy Age (year)	38.61 ± (1.11)	38.85 ± (1.24)	0.151
APGAR in 1 minute	8.94 ± (0.37)	8.96 ± (0.23)	0.602
APGAR in 5 minute	10.00 ± (0.00)	9.98 ± (0.17)	0.351
Weight of Neonate (g)	3206.87 ± (389.09)	3227.73 ± (430.40)	0.715
Lactogenic Medication	1.51 ± (0.50)	1.57 ± (0.50)	0.413
Weight of Neonate at 2 month age (g)	5048.73 ± (962.93)	5295.48 ± (803.95)	0.036

* Body Mass Index

**Table 2:** comparing categorical variables between spinal and non-spinal groups

**Variables**		**Spinal**	**Nonspinal**	**P-value** *
		**Count (%)**	**Count (%)**	
Job	Housewife	74(92.5%)	152 (89.4%)	0.499
Education grad	Primary school	22 (27.8%)	65 (38.7%)	0.228
	Primary to high school	48 (60.8%)	84 (50.0%0	
	University	9 (11.4%)	19 (11.3%)	
Breastfeeding History		35 (43.8%)	84 (49.4%)	0.419
Gender of Neonate	Male	44 (55.0%)	87 (51.2%)	0.590
	Female	36 (45.0%)	83 (48.8%)	
Ward	Mothers	72 (90.0%)	142 (83.5%)	0.246
	High Risk	8 (10.0%)	28 (16.5%)	
Diabetes mellitus or Gestational Diabetes		6 (7.5%)	15 (8.8%)	0.811
Lactogenic medication		39 (48.8%)	72 (42.6%)	0.413
Feeding rout in two month age	Breastfeeding	54 (67.5%)	121 (71.2%)	0.725
	formula	4 (5.0%)	10 (5.9%)	
	Combination of Breastfeeding and formula	22 (27.5%)	39 (22.9%)	
Completely formula feeding in two month age		4 (5.0%)	10 (5.1%)	0.518

* chi square test was used

There were 131 (52.4%) boys and 119 (47.6%) girls among the newborns. The weight of the babies ranged between 2500 and 4700 g. Infants born to 14.4% of the women were admitted in the neonatal high-risk ward. There was no significant difference between the age, Body Mass Index (BMI) and gravid of the mothers allocated in each group (P-value > 0.05). As for the infant, similarly, the 1^st^ and 5^th^ min APGAR, and weight at the time of birth was not statistically different in their groups (P-value > 0.05) (Tables 1 and 2).

Among studied women, 14 (5.6%) did used formula completely to feed their newborn at 2 months after breath. 175 (70%) of them breastfed their children whereas 61 of them (24.4%) gave their children baby formulas or a combination of breast milk and baby. These feeding routes were not statistically different significantly between those who had received spinal opioids and those without spinal opioids (P-value = 0.725), as regarding complete formula feeding two months after cesarean section (P-value = 0.518). But the infants weight at the 2nd month was significantly different between those who had received spinal opioids and those without spinal opioids (P-value = 0.036) (Tables 1 and 2).

There was no significant difference between the opioid analgesics administration route (spinal opioids, systemic opioids or none) and the rate of breastfeeding in the mothers, also (P-value = 0.773). About 111 (44.6%) of the mothers reported the use of different medications for increasing their milk during the one month follow up interval which was distributed similarly in two groups of study (P-value = 0.413).

Considering Feeding at two month of age (solely Breastfeeding or combination feeding as the event vs. formula) as the dependent variable; In multivariate analysis using Logistic Regression for adjusting factor consisting of Mother Age, BMI, Job, Education, Pregnancy Age, Breastfeeding History, Sex, 1 minute and 5 minute APGAR, Neonatal weight at birth, and Lactogenic medications; finally Sex (male to female) (P = 0.105; OR = 0.33; 95% CI 0.88- 1.26), and lactogenic medications (yes to no) (P = 0.064; OR = 0.28; 95% CI 0.07- 1.07) were remained in the model (Table 3).

**Table 3:** Independent variables which affect feeding at two month of age

	**P-value**	**Odd Ratio**	**95% CI (confidence interval)**
neonate gender	0.105	0.33	0.88-1.26
lactogenic medications use	0.064	0.28	0.07-1.07

**Table 4:** Independent variables which affect neonatal weight at two month of age

	**P-value**	**β**	**95% CI (confidence interval)**
Spinal vs. non-spinal anesthesia	0.009	327.12	84.08- 570.15
neonatal weight at birth	0.000	0.75	0.47- 1.03
lactogenic medications use	0.020	-276.99	43.51- 510.48

Considering neonatal weight at two month age as the dependent variable; In multivariate analysis using ANCOVA for adjusting factor consisting of Mother Age, BMI, Job, Education, Pregnancy Age, Breastfeeding History, Sex, 1 minute and 5 minute APGAR, Neonatal weight at birth, and Lactogenic medications; finally type of intervention (Spinal vs. non-spinal anesthesia) (P = 0.009; β = 327.12; 95% CI 84.08- 570.15), neonatal weight at birth (P = 0.000; β = 0.75; 95% CI 0.47- 1.03) and lactogenic medications (no vs. yes) (P = 0.020; β= -276.99; 95% CI 43.51- 510.48) remained in the model (Table 4).

## Discussion

Although breastfeeding has been shown to be beneficial to mother and the newborn, many women are unsuccessful in their attempts. The Department of Health Infant Feeding Survey, 2000, indicated that only 69% of women giving birth in the United Kingdom attempted to breastfeed, and 39% were exclusively bottle feeding on discharge from hospital (21). These values are clearly higher in our study group even in two months after birth. 

Breast-feeding problems can be due to infant (palate structure) or maternal issues (inverted nipples, lack of education); there are many factors that are thought to influence the process (22, 23). We tried to exclude such confounders as much as possible.

The use of pain killers has become increasingly popular for labor analgesia; however, their effects on breastfeeding remain unclear (24-25). Many studies have reported that opioids, particularly when used epidurally, cross the placenta and negatively influence the newborn’s neurobehavioral (6, 10, and 26). Others have reported that the pharmacological effects of these agents are responsible for the breastfeeding problem reported in these individuals (13).

There are, however, contradictory reports regarding the impact of consuming these opioids on breast feeding (6, 24). 

Several studies have pointed out the negative influence of epidural analgesia on breastfeeding duration, stressing that the consumption of higher doses of fentanyl, which has high lipophilic and easily crosses the placenta, can lead to neonatal depression and negatively affect breastfeeding (27- 29). Torvaldsen et al, for instance, reported that women who had epidurals were less likely to fully breastfeed their infant in the few days after birth and more likely to stop breastfeeding in the first 24 weeks (30). 

The strongest evidence to date comes from a recent randomized controlled trial in which 177 women who had previously breastfed were randomized to receive an epidural containing either no fentanyl, intermediate dose fentanyl (up to 150 μg fentanyl (or high dose fentanyl (> 150 μg fentanyl). Beilin et al reported that 65% of expectant mothers receiving more than 150μg of fentanyl epidurally are at a higher risk of nursing problems. They added that the risk is as low as 35% in those receiving lower doses of the drug (25).

Wittels et al, on the other hand, reported a lower consciousness level in babies born to women who have received meperidine than those receiving morphine through Patient Control Analgesia (PCA) device (31). This could be confirmatory to our results about morphine.

In line with the present study, three North American studies found no association between epidurals and breastfeeding duration (32-34). 

However, there are recently published reports about the safety of epidural or intrathecal opioids (17-19) and not obvious effect in the case of single dose systemic opioids (34). Even there is a report that suggesting successful lactation and safety for infant despite long term intrathecal morphine (35). Our result is also supporting these recent reports. 

The present study, for the first time, revealed that the use of spinal morphine does not have any impact on the breastfeeding.

Meanwhile the low educational profile of our patients may have affected our results. It is therefore recommended to perform further studies in a broader group of individuals.

It should be added that the present study also revealed that the number of gravid was the only factor influencing the impact of the administration of opioid route on the breastfeeding rate. Indicating that factors such as the maternal education, age, BMI, profession, the infant’s birth weight and the use of products believed to improve the nursing did not affect the association.

Based multivariate analysis using Logistic Regression test; male sexuality, and lactogenic medications were associated with more formula vs. sole Breastfeeding or combination feeding.

In multivariate analysis using regression; non-spinal group vs. Spinal, higher neonatal weight at birth had resulted higher weight at two month age, while lactogenic medications use was associated with lower weight at two month age.

It seem that lactogenic medications had been used when there was breastfeeding problems, but such medications did not effective and neonatal weight at two month age remain lower in this subgroup of patients. However, this hypothesis needs more studies to be documented.

Neonates with higher weight at birth had higher weight in 2 month age but the mean difference diminished. There is not a clear hypothesis by authors to discuss the reason to lower weight in intrathecal opioid group, despite the equal rate of breastfeeding. However, it is so important issue and should be investigated.

As a hypothesis and based on some social problems, higher rate of formula use in male infants may be the result of difference in sucking and behavioral characteristics related to gender or more probably related to gender discrimination by parents. This should be assessed in future studies.

In view of the fact that various factors including the psychosomatic, environmental, socio-economic and cultural issues influence the breastfeeding, it was impossible to address the effect of all these variables in this study. Moreover, it was not feasible to visit all the mothers at the end of the month to assess the quality and quantity of breastfeeding or evaluating the mother and infant for possible problems. It is suggested that cultural and socioeconomic characteristics of our cases, had interfered with access to patients in our study group and result in high percentage of excluded cases. As a result, it would be beneficial to assess all the above mentioned variables in a larger study.

## Conclusion

This is the first study which suggests that intrathecal morphine do not interfere with breastfeeding. The impacts of spinal anesthesia intrathecal morphine on weight gaining need more surveys to be clear.
